# Supplementary material for: Decision analysis of PPP project’s parties based on deep consumer participation
Source: PLoS One. 2024 Apr 16;19(4):e0299842. doi: 10.1371/journal.pone.0299842 (PMC11020698; doi:10.1371/journal.pone.0299842)
Supplement: S1 Data — (DOCX) [file pone.0299842.s001.docx]

**Minimal data**

## 4.1 Evolutionary path of ESS

Based on the replication dynamic equation of the tripartite evolutionary game, this section employ MATLAB to simulate the evolutionary path of the seven ESS points mentioned in section 3.

Assume ,$R=60,R_{g}=10,C_{g}=20,C_{H}=50,C_{L}=40,V=20,a=0.9,b=0.9,F=5,C_{s}=8$ ,the evolutionary path of (0, 0, 0) is demonstrated in Fig.1(a).

Assume ,$R=60,R_{g}=10,C_{g}=15,C_{H}=50,C_{L}=40,V=20,a=0.9,b=0.9,F=5,C_{s}=8$ ,the evolutionary path of (0, 1, 0) is demonstrated in Fig.2(a).

Assume ,$R=60,R_{g}=10,C_{g}=20,C_{H}=50,C_{L}=40,V=20,a=0.9,b=0.9,F=5,C_{s}=4$ ,the evolutionary path of (0, 0, 1) is demonstrated in Fig.3(a).

Assume ,$R=60,R_{g}=20,C_{g}=10,C_{H}=50,C_{L}=40,V=20,a=0.4,b=0.9,F=5,C_{s}=4$ ,the evolutionary path of (1, 1, 0) is demonstrated in Fig.4(a).

Assume ,$R=60,R_{g}=10,C_{g}=20,C_{H}=50,C_{L}=40,V=20,a=0.4,b=0.8,F=5,C_{s}=-3$ ,the evolutionary path of (1, 0, 1) is demonstrated in Fig.5(a).

Assume ,$R=60,R_{g}=20,C_{g}=10,C_{H}=50,C_{L}=40,V=20,a=0.8,b=0.8,F=5,C_{s}=$4,the evolutionary path of (0, 1, 1) is demonstrated in Fig.6(a).

Assume ,$R=60,R_{g}=20,C_{g}=10,C_{H}=50,C_{L}=40,V=20,a=0.4,b=0.8,F=5,C_{s}=-3$ ,the evolutionary path of (1, 1, 1) is demonstrated in Fig.7(a).

## 4.2. Impacts of key parameters on evolutionary results and trajectories

In order to further analyze the impact of some key parameters in the tripartite evolutionary game,a numerical simulation based on a scenario is conducted in this section. The initial values of all parameters based on the reality scenario are:$R=60,R_{g}=20,C_{g}=10,C_{H}=50,C_{L}=40,V=20$,a=0.9,b=0.9,F=5,$b_{1}=20$,$b_{2}=10,C_{s}=4$
