# Supplementary material for: Decision analysis of PPP project’s parties based on deep consumer participation
Source: PLoS One. 2024 Apr 16;19(4):e0299842. doi: 10.1371/journal.pone.0299842 (PMC11020698; doi:10.1371/journal.pone.0299842)
Supplement: Attachment — Submitted filename: PLOSONE.pdf [file pone.0299842.s002.pdf]

This paper studies a tripartite evolutionary game of government, producers, and consumers to study how infrastructural projects can be best promoted. It conducts MATLAB simulations of the model. The results are competently done.

However, the writing needs to be significantly improved. A native English-speaking copyeditor is suggested. I'm sorry to say, but it is a pain to read from the very first sentences.

In addition, the list of references needs to be better prepared. The numbering is duplicated and off. For example, in the screenshot below, the numbers are off, the names are not spelled out, and journals like Econometrica are included as part of the title. The problem arises from #10 on the reference list.

31. 30. D. Y. H. Y. Y. X. H. (2019). " Exploring the Moral Hazard Evolutionary Mechanism for BIM Implementation in an Integrated Project Team." Sustainability **11:5719**.
32. 31. S. H. Y. Z. L. Z. Z. (2019). "Evolutionary game of the green investment in a two-echelon supply chain under a government subsidy mechanism." J Clean Prod **235:1315–26**.
33. 32.Y. X. Z., J.; Shen G.Q.; Yan Y. (2019). "Incentives for green retrofits: An evolutionary game analysis on Public-Private-Partnership reconstruction of buildings." J. Clean. Prod **232**: 1076–1092.
34. 33. F., D (1991). "Evolutionary games in economics. Econometrica." **59 (3)**: 637–666.
35. 34.L., A.M (1992). "The general problem of the stability of motion." Int. J. Contr. **55(3)**:

In addition, there are missing discussions on related work:

"Decision model to optimize long-term subsidy strategy for green building promotion" in *Sustainable Cities and Society* also studies a tripartite evolutionary game model between government, producers, and consumers.

"Profit versus sustainability in bikeshare" in *Sustainable Cities and Society* studies sustainability of infrastructure projects.
