# Supplementary material for: Decision analysis of PPP project’s parties based on deep consumer participation
Source: PLoS One. 2024 Apr 16;19(4):e0299842. doi: 10.1371/journal.pone.0299842 (PMC11020698; doi:10.1371/journal.pone.0299842)
Supplement: Attachment — Submitted filename: Response to Reviewer.docx [file pone.0299842.s003.docx]

Dear Editors and Reviewers:

Thank you for your letter and for the reviewers’comments concerning our manuscript entitled “Decision Analysis of PPP Project’s Parties Based on Deep Consumer Participation” (ID:PONE-D-23-19775).Those comments are all valuable and very helpful for revising and improving our paper, as well as the important guiding significance to our researches. We have studied comments carefully and have made correction which we hope meet with approval.

***Reviewer #1: Please see attached. Most importantly, writing needs to be improved. Second, the reference list needs to be better organized.***

Response:I have checked the grammar and words in the whole text and made changes to make the sentences clearer, concise and readable.

***Please see attached. Most importantly, writing needs to be improved. Second, the reference list needs to be better organized.***

Response:I have checked the reference and reorganized it.

***Reviewer #2: This paper is interesting and find some valuable conclusion. The paper can be accepted after making the following revisions.***
***1. The innovation of this paper needs to be highlighted in the abstract.***

Response :I have made revisions to the abstract, highlighting the innovation of the article and emphasizing the significant impact of consumer's active involvement on decision-making processes within the project.

***2. The author introduced the research background of this paper too much, but they does not explain***

Response :The language in the background section has been reorganized, and unnecessary content has been removed.

***3. The literature review is not enough, the innovation of this paper and the contribution made by previous studies have not been clearly expressed. The innovation of this paper and the contribution made by previous studies have not been clearly expressed. The author should summarize the existing research gaps and highlight the innovation of this paper after completing the literature review.***

Response :I have reorganized the existing literature review by providing an overview of the content in the first sentence of each paragraph. Additionally, I have included research findings on consumer participation in various types of projects. Finally, I have summarized the literature review, highlighting existing research gaps and the innovation of this study.Thank you.

***4. The presentation of the method is very imprecise***

Response :I have reviewed the parameter expressions, sentence coherence, and spelling errors in the methods section. I have also reorganized the language and improved some expressions that were incorrect or omitted.

***5. Interpretation of results does not highlight important issues studied in this paper***

Response :The article has added a discussion section, which analyzes the impact of the variations in the main parameters on the three parties and showcases the theme of the paper. Additionally, the conclusion section has included the main conclusions of the article.

***6. The discussion section does not analyze the results of this paper, and provides enlightening thinking on related issues. the following literature should be helpful for your research：(1) Reduction pathways identification of Agricultural Water Pollution in Hubei Province, China. (2) Coordination of the Industrial-Ecological Economy in the Yangtze River Economic Belt, China.***

Response :The article has added a discussion section, which analyzes the impact of the variations in the main parameters on the three parties and showcases the theme of the paper.Thank for your help.

***7. Compared with the available literature, what are the theoretical contributions and application values of this study? It is suggested to enhance the corresponding discussions in the conclusion part.***

Response :I have added two paragraphs in the conclusion section that discuss the impact of consumer participation on PPP project governance and the contribution of this study to the theory.
